# Arabinoxylan from Corn Fiber Obtained through Alkaline Extraction and Membrane Purification: Relating Bioactivities with the Phenolic Compounds

**DOI:** 10.3390/molecules28155621

**Published:** 2023-07-25

**Authors:** Verónica Weng, Martim Cardeira, Andreia Bento-Silva, Ana Teresa Serra, Carla Brazinha, Maria Rosário Bronze

**Affiliations:** 1 LAQV-Requimte, Department of Chemistry, NOVA School of Science and Technology, NOVA University of Lisbon, 2829-516 Caparica, Portugal; v.weng@campus.fct.unl.pt; 2 ITQB NOVA, Instituto de Tecnologia Química e Biológica António Xavier, Universidade Nova de Lisboa, Avenida da República, 2780-157 Oeiras, Portugal; martim.cardeira@ibet.pt (M.C.); mbronze@ibet.pt (M.R.B.); 3 iBET, Instituto de Biologia Experimental e Tecnológica, Avenida da República, Quinta-do-Marquês, Estação Agronómica Nacional, Apartado 12, 2780-157 Oeiras, Portugal; 4 FFULisboa, Faculdade de Farmácia, Universidade de Lisboa, Avenida das Forças Armadas, 1649-019 Lisboa, Portugal; abentosilva@ff.ulisboa.pt; 5 iMed.ULisboa, Instituto de Investigação do Medicamento, Faculdade de Farmácia, Universidade de Lisboa, Avenida Professor Gama Pinto, 1649-003 Lisboa, Portugal

**Keywords:** arabinoxylan, ferulic acid, cytotoxicity, Caco-2 cell line, antiproliferative effect, HT29 cell line

## Abstract

Arabinoxylan has prebiotic properties, as it is able to resist digestion in the small intestine and undergoes fermentation in the large intestine. In this work, arabinoxylan was extracted from corn fiber using an alkaline solution and further purified with membrane processing. It was found that the extracts were mainly composed of xylose (50–52%), arabinose (37–39%), galactose (9%) and glucose (1–4%), with an A/X ratio of 0.72–0.77. All the extracts were composed of phenolic compounds, including ferulic acid derivatives such as dimers, trimers and tetramers. The purified extract had a lower concentration of ferulic and *p*-coumaric acid (0.004 and 0.02 mg/mg_dry_weight_, respectively) when compared to raw extract (19.30 and 2.74 mg/mg_dry_weight_, respectively). The same effect was observed for the antioxidant activity, with purified extracts having a lower value (0.17 ± 0.02 µmol TEAC/mg) when compared to the raw extract (2.20 ± 0.35 µmol TEAC/mg). The purified extract showed a greater antiproliferative effect against the HT29 cell line with EC_50_ = 0.12 ± 0.02 mg/mL when compared to the raw extract (EC_50_ = 5.60 ± 1.6 mg/mL). Both raw and purified extracts did not show any cytotoxicity to the Caco-2 cell line in the maximum concentration tested (10 mg/mL).

## 1. Introduction

Arabinoxylan is a non-starch polysaccharide and a major source of dietary fiber, meaning that it resists the digestion process in the human small intestine and undergoes partial or complete fermentation by microbiota in the human large intestine. Due to these features, arabinoxylan can be considered a prebiotic [1]. Prebiotic fermentation produces short-chain fatty acids such as acetic, propionic and butyric acid. The presence of these acids lowers the pH of the intestine, influencing the type of bacteria that are present in the intestine. Some pathogenic bacteria like *Salmonella*, *Escherichia coli* and *Clostridium* have shown difficulty to proliferate in acidic environments [2]. The production of butyric acid is also connected with the growth and differentiation of epithelial cells in the intestinal tract [2].

Arabinoxylan can be found in cereal grains such as corn, wheat, rye, barley, oats, rice and sorghum [3,4]. Its structure comprises a chain of linear linked *β*-(1,4)-d-xylopyranosyl residues, which can then be substituted by α-L-arabinofuranose units as side chains, and other minor substituents include galactose, glucose and mannose [3,5]. These arabinose units can be esterified (covalently bound) with hydroxycinnamic acids such as ferulic acid and, in a smaller quantity, by *p*-coumaric acid [3], also known as insoluble-bound phenolics.

Corn fiber is an abundant by-product obtained from the starch industry that is integrated into animal feeds or just discharged as waste. However, corn fiber is a rich and natural source of arabinoxylan, if extracted and purified properly. Besides arabinoxylan, corn fiber extract is also rich in ferulic acid, a crosslinker of arabinoxylan, and a known antioxidant and precursor of bio-vanillin [6,7]. The extraction method of arabinoxylan from corn fiber can influence its biological properties since it affects the final molecular weight, branching and ferulic acid content [5]. Alkaline extraction is reported as one of the most simple and economic methods to extract arabinoxylan [7]. More extreme conditions release phenolic compounds with a high yield from the polysaccharide. However, they also compromise its main arabinoxylan structure; therefore, mild conditions are more favorable to maintain its integrity and still obtain a good yield of phenolic compound extraction [8].

Cereal grains show different structural complexity, which may be evaluated by the degree of branching, also known as the degree of substitution. The degree of substitution in arabinoxylan can be described as the ratio of arabinose (A) substituents in relation to the xylose (X) units in the backbone, being represented as the A/X ratio. This important parameter is related to the properties of arabinoxylan, including biological properties, since it is related to its fermentation process. It was found that the degree of fermentation was lower when the A/X was higher, meaning that xylose molecules that are less substituted with arabinose are preferentially fermented. Likewise, highly feruloylated arabinoxylan is less fermented than low-feruloylated arabinoxylan, since the presence of ferulic acid can sterically hinder enzyme activities [3,9]. Cereals like wheat and rye have a less branched structure and complexity (A/X ratio = 0.5–0.7 and 0.48–0.55, respectively), in comparison to rice, corn and sorghum (A/X ratio = 0.8, 0.74 and 0.87, respectively), which have a more complex structure and branching [5,10].

The consumption of dietary fibers is related to various health benefits besides prebiotic activity, such as improving lipid and fat metabolism and reducing the risk of various diseases [9]. Arabinoxylan consumption, more precisely, has been associated with a reduction in the risk of chronic cardiovascular diseases, gastrointestinal cancer, type II diabetes and rheumatoid arthritis and prevention of the overgrowth of pathogenic bacteria [1,4,11].

Nevertheless, there are only a few studies evaluating the influence of arabinoxylan from corn fiber in terms of cytotoxicity and antiproliferative effect in human cell lines. In order to evaluate the possibility of applications in the pharmaceutical and food industries, it is mandatory to understand if arabinoxylan has any effect on growth/proliferation or shows any cytotoxicity towards human cell lines. The cytotoxicity of arabinoxylan (from dried distillers’ grains with solubles) was previously evaluated by studying its effect on the growth of normal human colon cells (cell line CCD 841 CoN) using the 3-(4,5-dimethylthiazol-2-yl)-2,5-diphenyltetrazolium bromide (MTT) assay and doxorubicin as a positive control. Different concentrations were tested (125, 250, 500 and 1000 μg/mL of arabinoxylan). The results showed that arabinoxylan did not affect the proliferation or morphology of CCD 841 CoN cells, independently of the concentration tested, and did not show any toxic effect on the normal human colon cell line tested [12]. The antiproliferative effect of the resulting fermentation supernatants under anaerobic conditions from wheat bran arabinoxylan, extracted in both water and alkali conditions, was studied using the HT29 cell line. It was found that all samples effectively slowed the growth of HT29 cells, and significant inhibitory effects were observed after 72 h at a concentration of 4% [13].

In the present work, a sustainable (economic and environmentally friendly) process is proposed to obtain a purified arabinoxylan fraction. This process involves a mild alkaline extraction and membrane processing, with no organic solvents, mild temperatures and low transmembrane pressures. Chemical characterization, cytotoxicity and antiproliferative activities were tested in the different arabinoxylan fractions obtained from corn fiber after each processing step of the extraction and purification processes in order to understand the relationship between the phenolic compounds present in each fraction and the cytotoxicity or bioactivity detected.

## 2. Results and Discussion

### 2.1. Extraction and Purification of Arabinoxylan from Corn Fiber

**Figure 1:**
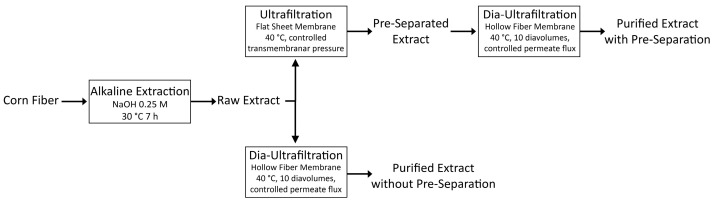
Scheme of the extraction and different purification methods, as well as conditions, of arabinoxylan originating from corn fiber and resulting extracts. All the details of each processing step are shown in Section 3.2.1 and Section 3.2.2.

Arabinoxylan was extracted from corn fiber and further purified through membrane processes using two methods. For a better understanding of the membrane methods used to obtain the extracts, a scheme illustrating the different processing steps is shown in Figure 1.

In both membrane methods, two final arabinoxylan-rich fractions were obtained, identified as Purified Extract with Pre-Separation and Purified Extract without Pre-Separation.

### 2.2. Characterization of Arabinoxylan Extracts

#### 2.2.1. Sugar Content and A/X Ratio of Arabinoxylan Extracts

Arabinoxylan extracts (raw, pre-separated and both purified) were characterized in terms of sugar content. They were mainly composed of xylose (50–52%) and arabinose (37–39%), and a small percentage of galactose (9%) and glucose (1–4%) were also found. The significant percentage of galactose found in the samples may be due to the raw material from which the arabinoxylan was extracted, since the outer layers of corn have a higher concentration of galactose [14]. Nevertheless, the percentages of the sugars found in the extracts are similar to those reported by Ward, 2020 [14]. The ratio of arabinose in relation to xylose (A/X ratio) was 0.72–0.77, which is also similar to what is reported by Knudsen, 2014 [10].

#### 2.2.2. Characterization of Phenolic Compounds though HPLC-DAD-MS/MS

The main phenolic compounds present in the extracts were identified based on a previous work (Bento-Silva et al., 2020) [15]. Ferulic and *p*-coumaric acid were found to be present, as well as dimers, trimers and tetramers of ferulic acid, some of which were bonded by amine compounds. Vanillin, *p*-hydroxybenzoic acid and *p*-hydroxybenzaldehyde were also identified (see Table S1, Figures S1 and S2). It was found that most compounds were removed during the purification process in diafiltration mode, with very few still being detected in the purified extracts.

Most compounds were effectively removed with only a purification step in diafiltration mode (purification without the pre-separation step). However, compounds like *p*-hydroxybenzoic acid, vanillin, *p*-coumaric acid and other more complex compounds were not detected only in the purified extract after the pre-separation step, meaning that this pre-step might aid in the effective removal of these compounds. However, some compounds such as *p*-hydroxybenzaldehyde and ferulic acid and its dimers, trimers and tetramers were still detected in the purified extract after the pre-separation step. These results show that some compounds were more easily removed during the purification process, since some of them were present in high amounts in the raw extract but were not detected in the purified extract, while others, present in lower amounts in the raw extract, were still present after the purification process (Table S1). This may be explained by some compounds still being connected to the arabinoxylan structure.

#### 2.2.3. Ferulic Acid and *p*-Coumaric Acid Contents and Antioxidant Activity (ORAC) of the Extracts Produced

The concentrations of ferulic acid and *p*-coumaric acid, which are dominant phenolic compounds in corn fiber, were determined using HPLC. Additionally, the antioxidant activity was determined through the oxygen radical absorbance capacity (ORAC) method in Trolox equivalent antioxidant capacity (TEAC) for all the extracts produced in the purification steps through membrane processing (see Figure 1), and these are summarized in Table 1.

The pre-separation step is operated in concentration mode, where no water is added to the retentate. Therefore, it becomes more viscous as the separation occurs, negatively influencing the efficiency of the process. The purified extract with pre-separation and purified extract without pre-separation, respectively preceded or not by the pre-separation step, are operated in continuous diafiltration mode, where water is added at a similar rate to that at which the permeate is collected, avoiding viscous retentate and more efficiently removing small compounds from the retentate, which becomes enriched in large arabinoxylan compounds. This mode avoids the obstruction of the membrane pores, which, overall, makes the efficiency of the process much more positive.

The results showed that the ultrafiltration operated in concentration mode is not as efficient at removing small compounds when compared with the ultrafiltration in diafiltration mode, as expected (extracts obtained from the diafiltration had 3 times lower concentrations of acids than the extract from the concentration mode, 4 and 5 × 10^−3^ mg/mg_dw_ vs. 25.13 mg/mg_dw_, respectively).

The antioxidant activity is most likely due to the presence diverse of hydroxycinnamic acids, such as ferulic and *p*-coumaric acid in solution; they are both known antioxidants and their antioxidant activity in terms of ORAC has been previously reported as 15.6 ± 1.10 and 23.0 ± 2.7 µmol TEAC/mg for ferulic and *p*-coumaric acid, respectively [7]. Other hydroxycinnamic acid derivates compounds like dicoumaroyl spermidine, an amide derivate, as well as dehydrodiferulic acid, a ferulic acid dimer, both still present in the purified extract with pre-separation (see Table S1), have been reported to contribute to the antioxidant activity of maize flours [16].This means that the lower the concentration of these acids in the solution, the lower the antioxidant activity. As expected, the purified extracts that had a lower concentration of ferulic and *p*-coumaric acid (they were also less rich in phenolic compounds) also presented the lowest antioxidant activity. The opposite happened with the raw extract and the pre-separated extract that have higher concentrations of both phenolic acids, which also reflects a higher value of antioxidant activity.

### 2.3. Cell-Based Assays

Cytotoxicity in the Caco-2-cell-line- and antiproliferative activity in the HT29-cell-line-based assays were tested for all arabinoxylan extracts. The results are presented in Table 2.

#### 2.3.1. Cytotoxicity in Caco-2 Cells

For the cytotoxicity assays, the Caco-2 cell line was chosen. These cells are epithelial-like cells from colon tissue derived from a patient with colorectal adenocarcinoma. However, they can be used as a healthy cell model since they replicate well the small intestine epithelium in a confluent/differentiation state [17]. This works well for our extracts since we want to study the potential prebiotic activity of arabinoxylan. Therefore, since this in vitro model is a well-studied and -known one, we are able to study how the cells perform when interacting with our extract.

As shown in Table 2, all samples did not reduce the viability of the Caco-2 cell line for the maximum concentration tested (10 mg/mL), meaning they were not cytotoxic. Moreover, it seems that the presence of phenolic compounds and other small compounds in the raw extract and pre-separated extract does not affect the viability of these cells.

#### 2.3.2. Antiproliferative Effect in HT29 Cells

For the antiproliferative assays, the HT29 cell line was used. These cells have an epithelial morphology and originated from a colorectal adenocarcinoma. They are commonly used for cancer and toxicology research and models, since they are sensitive to chemotherapeutic drugs used in treatments against colorectal cancer, such as 5-fluorouracil and oxaliplatin, which may be used as positive control [17,18].

In terms of antiproliferative effect, the purified extracts had the highest antiproliferative activity (lower EC_50_ values), followed by the pre-separated extract and the raw extract. Since the purified extracts had the more purified form of arabinoxylan (freed from other small compounds, like phenolics), the highest antiproliferative activity may be due to the presence of this bioactive compound rather than phenolic compounds. In fact, the extracts with the highest phenolic content (raw extract and pre-separated extract) (Table 1) were the ones with the lowest antiproliferative activity (Table 2). Arabinoxylan could be responsible for the antiproliferative effect of extracts as this non-starch polysaccharide has been suggested to help to induce apoptosis, a mechanism that plays a crucial role in preventing the proliferation of cancer cells [19,20]. Nevertheless, further studies are needed to unveil the mechanisms of action of arabinoxylan in colorectal cancer cells and also to identify other compounds still present in the purified extract that could also contribute to this bioactivity.

Glei et al., studied the effect of fermented supernatants in anaerobic conditions from alkali-extracted arabinoxylan in the proliferation of HT29 cells. It was observed that the growth of these cells was efficiently delayed at concentrations of 4% (*v*/*v*) [13].

## 3. Materials and Methods

### 3.1. Materials

For the extraction of arabinoxylan, NaOH (≥97% pellets, Sigma-Aldrich, St. Louis, MO, USA) was used. To purify the extract, two ultrafiltration membranes were used, a polyethersulfone (PES) with a flat sheet membrane configuration and a molecular weight cut-off (MWCO) of 150 kDa (Nadir UP150, Microdyn Nadir, Goleta, CA, USA) and a polysulfone with a hollow fiber membrane configuration and an MWCO of 100 kDa (UFP-100-C-5A, Cytiva, Marlborough, MA, USA). 2,20-azobis (2-methylpropionamidine)dihydrochloride (AAPH) and 20,70-dichlorofluorescein diacetate (DCFH-DA) were purchased from Sigma-Aldrich (St. Louis, MO, USA) for the ORAC assay. For cell-based assays, the Caco-2 (DSMZ, Braunschweig, Germany) and HT29 (ATCC, Manassas, VA, USA) cell lines, were used. Dulbecco’s modified Eagle’s medium (DMEM), Roswell Park Memorial Institute (RPMI) 1640 medium, non-essential amino acids (NEAA) and penicillin-streptomycin (Pen-Strep) were purchased from Invitrogen (Gibco, Paisley, UK). Heat-inactivated fetal bovine serum (FBS) was obtained from Biowest (Riverside, MO, USA). Phosphate-buffered saline (PBS) was purchased from Sigma-Aldrich (St. Louis, MO, USA). CellTiter 96 Aqueous OneSolution Cell Proliferation Assay (MTS) was obtained from Promega (Madison, WI, USA). For sugar composition analysis, trifluoroacetic acid (99%, Sigma-Aldrich, St. Louis, MO, USA) was used.

### 3.2. Experimental Procedure

#### 3.2.1. Arabinoxylan Extraction from Corn Fiber

Arabinoxylan was extracted from corn fiber, supplied by Copam-Companhia Portuguesa De Amidos, S.A., through a previous optimized process as described by Valério et al., 2021 and Serra et al., 2020 [21,22], using a solution of 0.25 M NaOH at 30 °C for 7 h with stirring. The final mixture was then centrifuged at 9174× *g* for 30 min. The solids were discarded and the supernatant was recovered, resulting in the raw extract.

#### 3.2.2. Arabinoxylan Purification with Membrane Processes

Raw arabinoxylan extract was purified through membrane processes in two different ways. The first was a process comprising a pre-separation step with a crossflow ultrafiltration process in concentration mode (initial feed concentrated 5 times) at 40 °C, under controlled transmembrane pressure conditions, followed by a purification step using a hollow fiber membrane, in continuous dia-ultrafiltration mode (where water is added to the feed solution at a similar rate to the permeate), for 10 diavolumes (a diavolume is reached when the volume of permeate collected is the same as the total volume present in the feed solution) at 40 °C and controlled permeate flux (the initial extract was diluted 10 times in order to be able to be processed in the membrane module). The other process was very similar to the previous one described, with the only difference being skipping the pre-separation process.

In both processes, the retentate fraction (arabinoxylan rich fraction) was recovered and the permeate fraction (with phenolic compounds) was used for other applications [23].

### 3.3. Analytical Methods

#### 3.3.1. Sugar Composition

Solutions of the different extracts were prepared by either weighing or diluting to a final concentration of around 1 mg/mL. Next, 5 mL of the solutions were transferred to a digestion tube and 100 μL of trifluoroacetic acid was added. The tubes were left at 100 °C for at least 4 h. The resulting solution was then filtered through a 0.15 μm filter and analyzed using HPLC. HPLC data were obtained by the Analysis Laboratory LAQV REQUIMTE—Chemistry department, FCT NOVA—Portugal, with a Dionex ICS3000 (Sunnyvale, CA, USA) equipped with a Thermo Carbopac PA10 250 × 4.6 mm (Waltham, MA, USA) column and a Thermo AminoTrap 50 × 4.6 mm (Waltham, MA, USA) pre-column. Samples were eluted with 18 mM NaOH (washed with 200 mM NaOH) at a flow rate of 1 mL/min at 25 °C and detected with ED—pulsed amperometric detection.

#### 3.3.2. Determination of Ferulic Acid and *p*-Coumaric Acid Using HPLC

Samples were analyzed through HPLC using a Dionex Ultimate 3000 equipment setup (Thermo Scientific, Waltham, MA, USA) equipped with an auto-sampler, pump and DAD. The separation of the compounds was performed on a reversed-phase column (Phenomenex, Luna RP18 100 Å (250 × 4 mm) 5 μm) at 35 °C, using an injection volume of 20 µL. The mobile phase consisted of 0.5% HCOOH/water as eluent A and 90% ACN + 0.5% HCOOH as eluent B at a flow rate of 0.6 mL/min for a total of 50 min (the elution gradient used was as follows: 94.4% of A and 5.6% of B from 0 to 15 min, 80% of A and 20% of B from 15 to 22 min, 60% of A and 40% of B from 22 to 45 min and 0% of A and 100% of B from 45 to 50 min). DAD was used to scan wavelength absorption at 280 nm.

#### 3.3.3. Antioxidant Activity through Oxygen Radical Absorbance Capacity (ORAC) Assay

The ORAC assay was performed to assess the antioxidant activity of each sample, following the method developed by Huang et al. [24], with minor modifications as reported previously [25]. This method measures the capacity of the samples to protect the disodium fluorescein (FL) from oxidation by peroxyl radicals (ROO^•^). Briefly, 150 µL disodium fluorescein (0.3 µM) was added to 25 µL of sample dilutions and incubated for 10 min at 37 °C in a black 96-well microplate. Subsequently, the reaction was initiated by the addition of 25 µL of 2,20-Azobis (2-amidinopropane) dihydrochloride (AAPH, 153 µM), and fluorescence (Ex/Em 485 ± 20/528 ± 20 nm) was measured for 40 min at 37 °C using a fluorescence microplate reader (FLx800 Bio-Tek Instruments, Winooski, VT, USA). A standard curve was prepared using 5, 10, 20, 30 and 40 µM of (6-hydroxy-2,5,7,8-tetramethylchroman-2-carboxylic acid (Trolox, Totowa, NJ, USA)). All solutions were prepared in phosphate-buffered saline (PBS), 75 mM, pH 7.4. The results are expressed as micromoles of Trolox equivalent antioxidant capacity per milligram of sample (µmol TEAC/mg dry sample).

#### 3.3.4. Characterization of the Major Compounds Using HPLC-DAD-MS/MS

Samples were analyzed using a Waters Alliance 2695 (Waters^®^, Dublin, Ireland) equipped with a quaternary pump, solvent degasser, auto sampler and column oven, coupled to a Photodiode Array Detector Waters 996 PDA and a Micromass^®^ Quattro Micro triple quadrupole (Waters^®^, Dublin, Ireland). The separation of the compounds was performed on a reversed-phase column (LiCrospher^®^ 100 RP-18, 250 × 4 mm; 5 µm; Merck^®^, Rahway, NJ, USA) at 35 °C, using an injection volume of 20 µL. The mobile phase consisted of Milli-Q water containing 0.1% formic acid (A): acetonitrile (B) at a flow rate of 0.30 mL/min, following the method described in Bento-Silva et al., 2020. DAD was used to scan wavelength absorption from 210 to 600 nm. Tandem mass spectrometry (MS/MS) detection was performed using an electrospray ionization (ESI) source operating at 120 °C, applying the conditions previously described (Bento-Silva et al., 2020) [15].

### 3.4. Cell-Based Assays

#### 3.4.1. Cell Culture

The human colorectal adenocarcinoma cell lines Caco-2 and HT29 were cultured in standard Dulbecco’s modified Eagle’s medium (DMEM) supplemented with 10% (*v*/*v*) fetal bovine serum (FBS), 1% (*v*/*v*) non-essential amino acids (NEAA) and 1% (*v*/*v*) penicillin-streptomycin, and Roswell Park Memorial Institute medium (RPMI) supplemented with 10% (*v*/*v*) FBS, respectively. Cells were routinely maintained as monolayers in 75 cm^2^ culture flasks and incubated at 37 °C with 5% CO_2_ in a humidified atmosphere.

#### 3.4.2. Cytotoxicity Assays

Cytotoxicity assays were performed using confluent and non-differentiated Caco-2 cells. Briefly, cells were seeded at a density of 2 × 10^4^ cells per well in 96-well plates, with medium (DMEM + 10% FBS, 1% PenStrep and 1% NEAA) renewal every 3 days. After 7 days of culture, the cells were confluent and were incubated for 72 h with the extracts at different concentrations diluted in culture medium (DMEM + 0.5% FBS + 1% NEAA). Wells containing cells incubated only with culture medium supplemented with 0.5% (*v*/*v*) of FBS were used as control. After 72 h incubation, the medium was removed, cells were washed with PBS and cell viability was evaluated using a CellTiter 96 AQueous One Solution Cell Proliferation Assay (MTS, Promega, Madison, WI, USA), according to the manufacturer. Absorbance was measured at 490 nm using an EPOCH2 microplate reader (Biotek Instruments, Winooski, VT, USA) and cell viability was calculated relative to the control. Three independent experiments were performed in triplicate.

#### 3.4.3. Antiproliferative Assays

The antiproliferative effect of extracts was evaluated in human colorectal adenocarcinoma HT-29 cells as previously described [26]. Briefly, cells were cultured in 96-well culture plates at a density of 1 × 10^4^ cells/well in RPMI supplemented with 10% (*v*/*v*) FBS. After 24 h, cells were incubated with different concentrations of the samples diluted in the culture medium (RPMI + 0.5% FBS) for 72 h. Cells incubated with only culture medium were considered as control. Cell proliferation was measured using MTS reagent, as mentioned previously. The results were expressed in terms of percentage of viable cells in comparison to the control. Three independent experiments were performed in triplicate.

## 4. Conclusions

In the present study, arabinoxylan was extracted with an alkaline solution and purified using various membrane processes, which produced a pre-separated extract and a purified extract obtained with and without a pre-separation step. The extracts were mainly composed of xylose (50–52%) and arabinose (37–39%), with an A/X ratio of 0.72–0.77. The extracts were also rich in various compounds, such as ferulic acid and its derivates including dimers, trimers and tetramers. The quantification of both ferulic and *p*-coumaric acid showed that the purification processes were effective in removing both compounds from the raw extract. The purified extract obtained using the pre-separation had a concentration of 4 × 10^−3^ and 2 × 10^−2^ mg/mg_dry_weight_ of ferulic and *p*-coumaric acid, respectively. The concentration for the purified extract obtained without the pre-separation step was 5 × 10^−3^ and 3 × 10^−3^ mg/mg_dry_weight_, respectively. The antioxidant activity may be derived from the presence of these phenolic acids; purified extracts had values of 0.17 ± 0.02 and 0.25 ± 0.04 μmol TEAC/mg, for purified extract with and without pre-separation, respectively. None of the extracts showed cytotoxicity to the Caco-2 cell line. In terms of antiproliferative effect, the purified extracts showed an interesting value of 0.12 ± 0.02 mg/mL and 0.29 ± 0.09 mg/mL for extracts with and without pre-separation, respectively.

## Figures and Tables

**Table 1:** Concentration of ferulic and *p*-coumaric acid (mg/mg_dry_weight_) and antioxidant activity determined through ORAC assay (µmol TEAC/mg) of raw extract, pre-separated extract and purified extract with and without pre-separation.

Sample	[FA] ^1^ (mg_[FA]_/mg_dw_^3^)	[CA] ^2^ (mg_[CA]_/mg_dw_^3^)	ORAC (µmol TEAC/mg_dw_^3^)
Raw Extract	19.30	2.74	2.20 ± 0.35
Pre-Separated Extract (retentate of the ultrafiltration UF, operated in concentration mode)	25.13	1.58	2.32 ± 0.45
Purified Extract with Pre-Separation (retentate of the UF, operated in diafiltration mode)	4.0 × 10^−3^	2.0 × 10^−2^	0.17 ± 0.02
Purified Extract without Pre-Separation (retentate of the UF, operated in diafiltration mode)	5.0 × 10^−3^	3.0 × 10^−3^	0.25 ± 0.04

^1^ [FA]: ferulic acid. ^2^ [CA]: *p*-coumaric acid. ^3^ dw: dry weight.

**Table 2:** Cytotoxicity (IC_50_) in Caco-2 and antiproliferative activity (EC_50_) in HT29 cell lines of raw extract, pre-separated extract and purified extracts with and without pre-separation.

Sample	IC_50_ (mg/mL) in Caco-2	EC_50_ (mg/mL) in HT29
Raw Extract	>10	5.60 ± 1.6
Pre-Separated Extract	>10	3.30 ± 0.37
Purified Extract with Pre-Separation	>10	0.12 ± 0.02
Purified Extract without Pre-Separation	>10	0.29 ± 0.09

## Data Availability

Not applicable.

## Supplementary Materials

The following supporting information can be downloaded at: https://www.mdpi.com/article/10.3390/molecules28155621/s1, Table S1: Putative identification of the compounds present in the raw, pre-separated and purified with and without pre-separation step extracts. Figure S1: Chromatogram of raw extract versus purified extract without pre-separation. Figure S2: Chromatogram of raw extract versus pre-separated extract and purified extract with pre-separation.
