# Supplementary material for: Arabinoxylan from Corn Fiber Obtained through Alkaline Extraction and Membrane Purification: Relating Bioactivities with the Phenolic Compounds
Source: Molecules. 2023 Jul 25;28(15):5621. doi: 10.3390/molecules28155621 (PMC10420191; doi:10.3390/molecules28155621)
Supplement: Supplementary file 1 [file molecules-28-05621-s001.zip › molecules-2502719-supplementary.pdf]

Table S1. Putative identification of the compounds present in the raw, pre-separated, and purified with and without pre-separation step extracts.

#ID	RT(min) [1]	Raw Extract	Purified extract without Pre-Separation	Pre-Separated Extract	Purified Extract with Pre-Separation	[M+H]+	[M-H]-	Putative Identification
**1**	36,95	✓	✓	✓	×	-	137	*p*-Hydroxybenzoic acid
**2**	49,03	✓	✓	✓	✓	-	121	*p*-Hydroxybenzaldehyde
**3**	59,35	✓	✓	✓	×	153	-	Vanillin
**4**	62,87	✓	✓	✓	×	-	163	*p*-Coumaric acid (*trans*)
**5**	67,67	✓	×	✓	×	603	-	*N,N'*-Coumaroyl dehydrodiferuloyl putrescine 1
**6**	71,97	✓	✓	✓	✓	-	193	Ferulic acid (*trans*)
**7**	75,10	✓	×	✓	×	-	595	TFA, hydrated 1
**8**	75,50	✓	✓	✓	×	-	385	8-8'-Dehydrodiferulic acid (cyclic)
**9**	79,31	✓	✓	✓	✓	-	403	Dehydrodiferulic acid, hydrated 1
**10**	80,00	✓	×	✓	×	-	403	Dehydrodiferulic acid, hydrated 2
**11**	80,57	✓	✓	✓	×	-	403	8-8'-Dehydrodiferulic acid (tetrahydrofuran)
**12**	81,45	✓	×	✓	×	-	403	Dehydrodiferulic acid, hydrated 3
**13**	83,00	✓	✓	✓	✓	-	403	Dehydrodiferulic acid, hydrated 4
**14**	83,68	✓	✓	✓	×	-	385	8-5'-Dehydrodiferulic acid
**15**	83,90	✓	✓	✓	×	-	577	Dehydrotriferulic acid 1
**16**	85,50	✓	×	✓	×	603	601	*N,N'*-Coumaroyl dehydrodiferuloyl putrescine 2
**17**	86,20	✓	✓	✓	×	-	577	Dehydrotriferulic acid 2
**18**	88,60	✓	×	✓	×	438	436	*N,N'*-Dicoumaroyl spermidine (*cis/cis*)
**19**	89,52	✓	×	✓	×	457	-	*N*-Dehydrodiferuloyl putrescine 1
**20**	92,30	✓	✓	✓	✓	438	436	*N,N'*-Dicoumaroyl spermidine (*cis/trans*)
**21**	92,35	✓	✓	✓	×	-	595	Dehydrotriferulic acid, hydrated 2
**22**	92,44	✓	✓	×	×	468	-	*N,N'*-Coumaroyl feruloyl spermidine (*cis/cis*)

**23**	92,52	✓	×	✓	×	441	-	*N,N'*-Diferuloyl putrescine (*cis/cis*)
**24**	93,43	✓	✓	✓	×	-	577	Dehydrotriferulic acid 3
**25**	94,40	✓	✓	✓	×	-	577	Dehydrotriferulic acid 4
**26**	95,48	✓	×	✓	×	468	-	*N,N'*-Coumaroyl feruloyl spermidine (*cis/trans*)
**27**	95,70	✓	×	✓	×	457	-	*N*-Dehydrodiferuloyl putrescine 2
**28**	96,00	✓	✓	✓	✓	-	595	Dehydrotriferulic acid, hydrated 3
**29**	97,00	✓	✓	✓	✓	438	436	*N,N'*-Dicoumaroyl spermidine (*trans/trans*)
**30**	97,25	✓	×	✓	×	-	577	Dehydrotriferulic acid 5
**31**	98,40	✓	✓	✓	✓		595	Dehydrotriferulic acid, hydrated 4
**32**	98,86	✓	×	✓	×	411	-	*N,N'*-Coumaroyl feruloyl putrescine (*cis, trans*)
**33**	98,88	✓	×	✓	×	468	-	*N,N'*-Coumaroyl feruloyl spermidine (*trans/trans*)
**34**	98,90	✓	×	✓	×	457	-	*N*-Dehydrodiferuloyl putrescine 3
**35**	99,34	✓	✓	✓	✓		595	TFA, hydrated 5
**36**	99,95	✓	×	×	×	-	769	Dehydrotetraferulic acid 1
**37**	99,95	✓	×	×	×	468	-	*N,N'*-Coumaroyl feruloyl spermidine (*trans/trans*)
**38**	100,00	✓	✓	✓	×	-	577	Dehydrotriferulic acid 6
**39**	100,02	✓	✓	✓	✓	-	385	5-5-Dehydrodiferulic acid
**40**	100,40	✓	✓	✓	✓	-	595	Dehydrotriferulic acid, hydrated 6
**41**	100,92	✓	×	✓	×	603	-	*N,N'*-Coumaroyl dehydrodiferuloyl putrescine 3
**42**	101,02	✓	×	✓	×	381	-	*N,N'*-Dicoumaroyl putrescine
**43**	101,54	✓	×	✓	×	441	-	*N,N'*-Diferuloyl putrescine (*cis/trans*)
**44**	102,29	✓	×	✓	×	603	-	*N,N'*-Coumaroyl dehydrodiferuloyl putrescine 4

**45**	102,99	✓	×	✓	×	603	-	*N,N'*-Coumaroyl dehydrodiferuloyl putrescine 5
**46**	103,06	✓	×	✓	×	411	409	*N,N'*-Coumaroyl feruloyl putrescine (*trans, trans*) 1
**47**	103,80	✓	×	✓	×	-	409	*N,N'*-Coumaroyl feruloyl putrescine (*trans, trans*) 2
**48**	104,57	×	×	✓	×	603	-	*N,N'*-Coumaroyl dehydrodiferuloyl putrescine 6
**49**	104,80	×	×	✓	×	-	769	Dehydrotetraferulic acid 2
**50**	105,33	✓	×	✓	×	441	439	*N,N'*-Diferuloyl putrescine (*trans/trans*)
**51**	105,40	✓	✓	✓	✓	-	385	8-O-4-Dehydrodiferulic acid (*trans/trans*)
**52**	105,92	✓	×	✓	×	603	-	*N,N'*-Coumaroyl dehydrodiferuloyl putrescine 7
**53**	106,00	✓	✓	✓	✓	-	385	8-5'-Dehydrodiferulic acid (bezofuran)
**54**	106,00	✓	×	✓	×	603	-	*N,N'*-Coumaroyl dehydrodiferuloyl putrescine 8
**55**	106,20	✓	✓	✓	×	-	385	8-O-4-Dehydrodiferulic acid (*trans/cis*)
**56**	106,44	✓	×	×	×	825	-	*N,N'*-Feruloyl dehydrotriferuloyl putrescine or *N,N'*-Didehydrodiferuloyl putrescine 1
**57**	108,00	✓	×	✓	×	-	769	Dehydrotetraferulic acid 3
**58**	109,82	✓	✓	✓	×	-	385	Dehydrodiferulic acid (other)
**59**	110,97	✓	✓	✓	✓	579	577	Dehydrotriferulic acid 7
**60**	113,38	✓	×	✓	×	825	-	*N,N'*-Feruloyl dehydrotriferuloyl putrescine or *N,N'*-Didehydrodiferuloyl putrescine 2
**61**	116,40	✓	×	✓	×	-	577	Dehydrotriferulic acid 8

**62**	117,80	✓	×	✓	×	633	-	*N,N'*-Feruloyl dehydrodiferuloyl putrescine 1
**63**	118,26	✓	×	×	×	603	-	*N,N'*-Coumaroyl Dehydrodiferuloyl putrescine 9
**64**	118,33	✓	×	✓	×	825	-	*N,N'*-Feruloyl dehydrotriferuloyl putrescine or *N,N'*-Didehydrodiferuloyl putrescine 3
**65**	119,10	✓	×	✓	×	879	877	*bis*-*N,N*'-Diferuloyl putrescine
**66**	121,00	✓	×	✓	×	-	769	Dehydrotetraferulic acid 4
**67**	121,38	✓	✓	✓	✓	579	577	Dehydrotriferulic acid 9
**68**	121,70	✓	×	✓	×	603	-	*N,N'*-Coumaroyl Dehydrodiferuloyl putrescine 10
**69**	122,20	✓	×	✓	×	825	-	*N,N'*-Feruloyl dehydrotriferuloyl putrescine or *N,N'*-Didehydrodiferuloyl putrescine 4
**70**	123,56	✓	✓	✓	✓	-	769	Dehydrotetraferulic acid 5
**71**	126,16	×	×	✓	×	825	-	*N,N'*-Feruloyl dehydrotriferuloyl putrescine or *N,N'*-Didehydrodiferuloyl putrescine 5
**72**	127,03	✓	✓	✓	✓	633	631	*N,N'*-Feruloyl dehydrodiferuloyl putrescine 2

[1] RT-Retention Time.

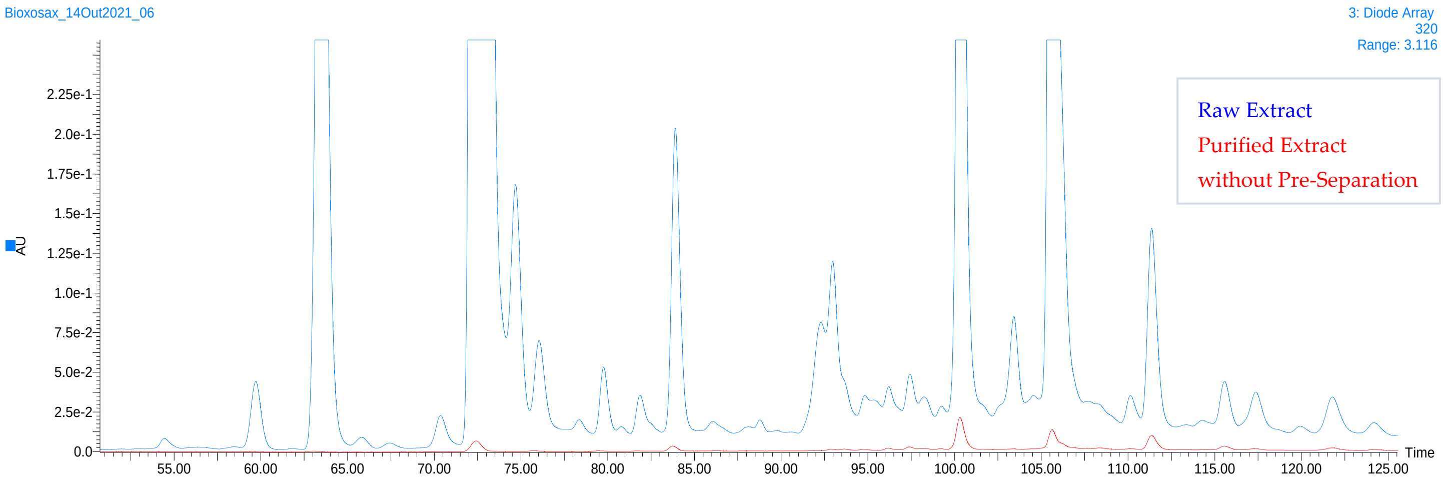

Figure S1. Chromatogram of raw extract *versus* purified extract without pre-separation.

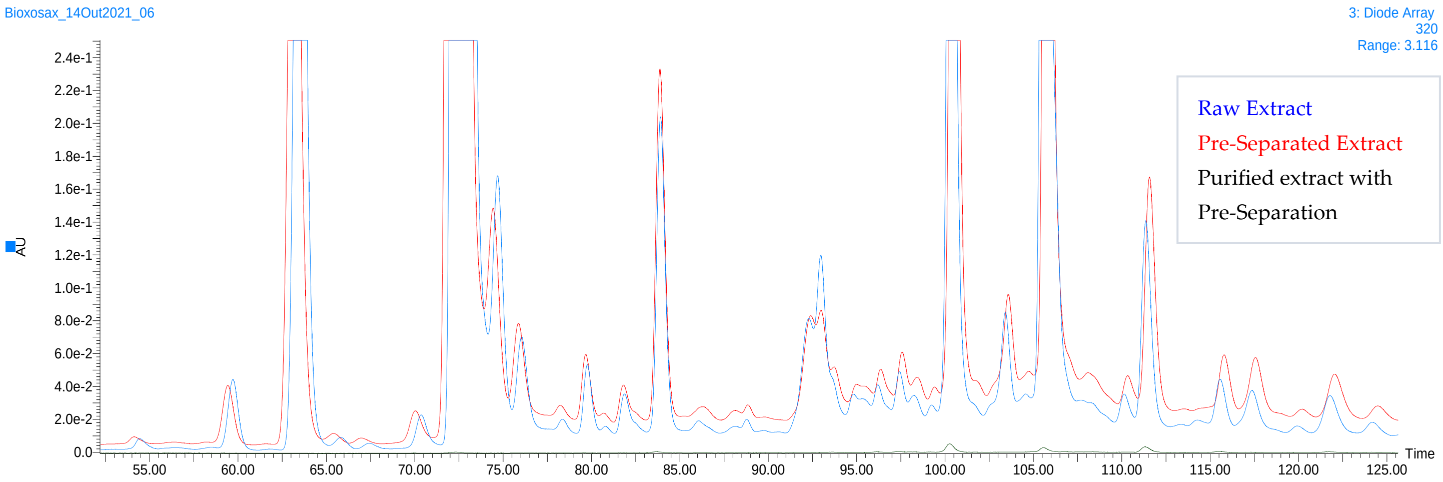

Figure S2. Chromatogram of raw extract *versus* pre-separated extract and purified extract with pre-separation.
